# Additive Wax-Up and Diagnostic Mockup As Driving Tools for Minimally Invasive Veneer Preparations

**DOI:** 10.7759/cureus.27402

**Published:** 2022-07-28

**Authors:** Jose Villalobos-Tinoco, Carlos A Jurado, Silvia Rojas-Rueda, Nicholas G Fischer

**Affiliations:** 1 Periodontics, National University of Rosario School of Dentistry, Rosario, ARG; 2 Prosthodontics, Woody L. Hunt School of Dental Medicine, El Paso, USA; 3 Dentistry, Universidad Javeriana, Bogota, COL; 4 Dentistry, University of Minnesota, Minneapolis, USA

**Keywords:** esthetic, ceramic, mock-up, dental procedures, veneer, wax-up

## Abstract

This report describes the importance of and outlines steps for additive wax-up and diagnostic mockup for anterior teeth as diagnostic and driving tools for non-prep and minimally invasive veneer preparations. A 35-year-old male presented to the clinic with the chief complaint of spaces between his front teeth. Diagnostic additive wax-up provided the possibility of offering minimally invasive preparations, and the use of a diagnostic intraoral mockup fulfilled the patient’s esthetic demands for treatment approval. Veneer preparations over the diagnostic mockup were provided as they are minimally invasive. Ceramic veneers were hand-crafted following the previous diagnostic wax-up, and restorations were bonded under rubber dam isolation. Overall, additive wax-up provides information needed to know if minimally invasive veneer preparations are possible, and the diagnostic mockup displays a physical, tentative outcome for the patient’s evaluation before irreversible tooth preparations. These simple, but effective, techniques can drive the diagnosis and prognosis of minimally invasive veneer restorations.

## Introduction

Dentistry was initially limited to treating caries and improving the function of and repairing tissues that were destroyed due to disease or trauma [1]. However, younger populations are distinctly interested in esthetic dentistry and patients are more aware of their anterior front teeth displayed in their smiles [2]. Indeed, some patients even give preference to treating teeth in the esthetic zone and do not mind leaving edentulous spaces in posterior areas [3]. A patient's self-esteem decreases when unsightly problems affect the smile, which leads to patients seeking care [4,5]. Esthetic dentistry is therefore in high demand at dental clinics.

Veneers are the most conservative treatment option for maintaining as much tooth structure as possible whenever teeth need to be treated for esthetic defects. Veneer preparations reduce 25% to 50% tooth structure in comparison to the traditional full-coverage crowns, so whenever possible, a veneer preparation is the recommended option [6]. While Veneers can be provided directly with resin composite or indirectly with ceramic materials, however, ceramic veneers have shown longer survival rates and better esthetic results [7]. Currently, many types of ceramic veneer materials are available on the market such as feldspathic porcelain, lithium disilicate, leucite, and zirconia, leading to many options for the clinician to address different clinician situations [8].

Clinicians are required to thoroughly evaluate many facial and dental parameters such as facial midline, smile line, tooth morphology, the position of lips, gingival tissues, the shade of teeth, and the number of displayed teeth to achieve optimal esthetic veneer restorations [9]. The diagnosis is key to evaluating if a patient is a suitable candidate for minimally invasive or traditional veneer restorations. Diagnostic wax-ups and intraoral mockups provide the information needed to evaluate how much tooth structure needs to be removed and the potential outcome of the proposed restorations [10,11]. Diagnostic wax-up can be done with two different techniques. The first is the traditional technique where the teeth in the cast are modified to place the wax and provide ideal contour, and the second is where the cast is not modified because the teeth have an ideal position and only a small amount of wax is placed on the cast teeth [10]. On the other hand, intraoral mockup is the transfer of the diagnostic wax-up to the mouth with a putty index and bis-acrylic material. This mockup provides a tactile evaluation of the proposed restorations [11]. At this stage, the patient can approve the treatment and the clinician can start with the tooth preparations [12]. Conservative tooth preparations are performed through the mockup with depth vertical reduction grooves on the incisal edge and horizontal reduction grooves on the facial surface to obtain the desired space for the ceramic restoration. Tooth preparation on the mockup provides relatively conservative reduction as it will reduce the bis-acrylic before the tooth structure and will be kept in an enameled structure. Once the preparation is finished, the remaining material is removed, the tooth is polished, and a final impression is provided [13]. This report aims to describe the steps for additive diagnostic wax-up and intraoral mockup as driving tools for the clinician to diagnose and offer minimally invasive veneer preparations to patients.

## Case presentation

**Figure 1:**
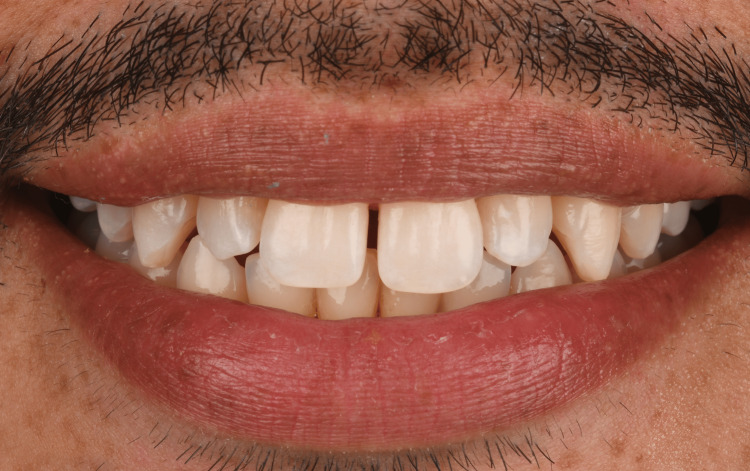
Initial smile of the patient

A 35-year-old male patient presented to the clinic with the chief complaint of having spaces between his front teeth (Figure 1). 

**Figure 2:**
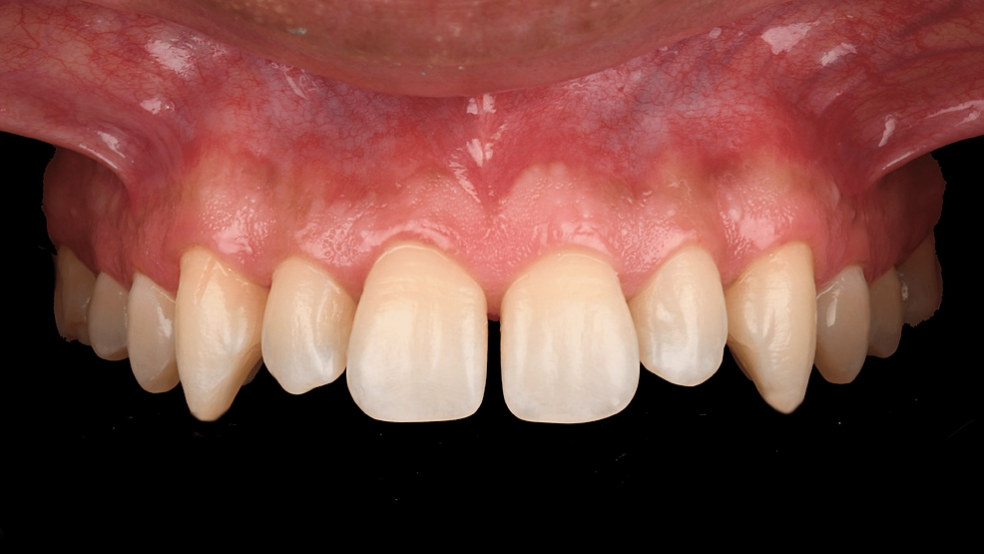
Initial intraoral presentation

Upon clinical evaluation, the patient was diagnosed with diastema between #5-6, 6-7, 7-8, 8-9, 9-10, 10-12, and 12-13 (Figure 2). 

**Figure 3:**
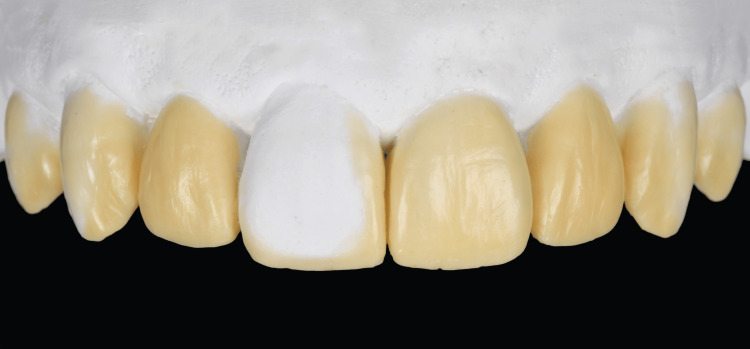
Diagnostic additive wax-up

Even though the patient had a low smile line that did not show the gingiva, the clinicians informed the patient of the non-ideal zenith position of the anterior teeth and periodontal surgery was offered for ultimately better esthetic results. However, the patient disliked the option of surgery and requested to have a conservative dental treatment with minimal dental anesthesia even though he did not have any contraindications. The patient was informed of the option of minimally invasive veneer restorations but that it would require a detailed intraoral evaluation, diagnostic wax-up, and mockup to see if he is a candidate for those types of restorations. The patient accepted and requested to start the minimally invasive process. Diagnostic impressions were made with polyvinyl siloxane (Virtual, Ivoclar Vivadent, Liechtenstein, Germany) material and poured out with type IV stone (Fujirock, GC America). Diagnostic casts were mounted in a semi-adjustable articulator (Artex CR, Amann Girrbach, Herrschaftswiesen, Austria) with a facebow record and a diagnostic wax-up (Wax GEO Classic, Renfert, Hilzingen, Germany) was performed without modifying the cast (Figure 3). 

**Figure 4:**
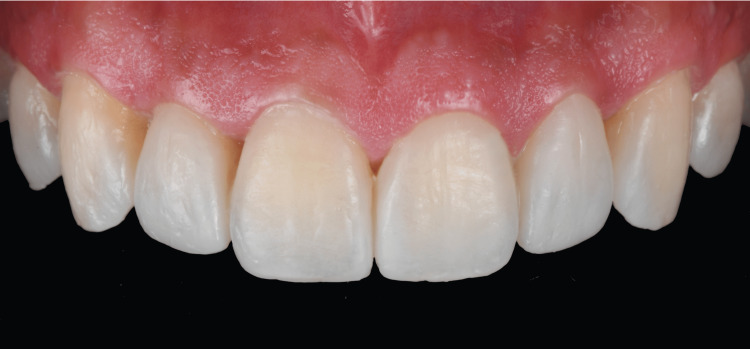
Diagnostic mockup

A putty matrix and light body impression material (Elite P&P, Zhermack, Rovigo, Italy) were placed on the diagnostic wax-up in order to fabricate the index guide for the diagnostic mockup. The putty guide was filled up with self-curing bis-acrylic (Telio CS C&B, Ivoclar Vivadent) and placed on the patient’s mouth to transfer the size, shape and all contours provided in the diagnostic mockup (Figure 4).

**Figure 5:**
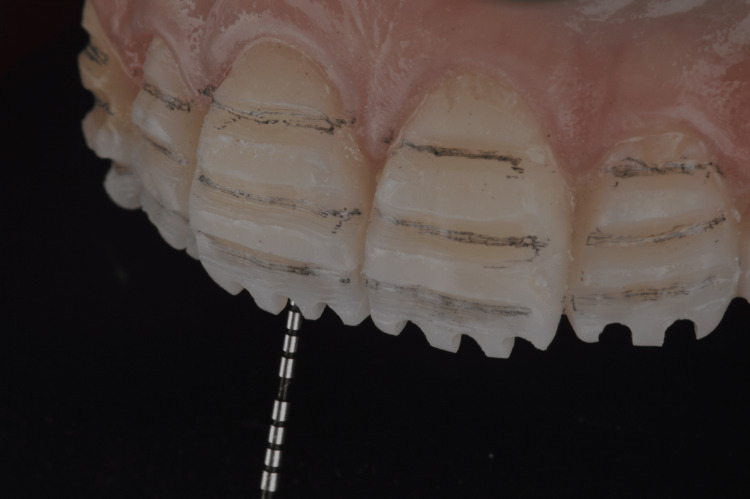
Conservative tooth preparations

The patient evaluated the intraoral mockup and requested to continue with the veneer restorations from tooth #5 to #12. Tooth reduction guides were fabricated based on the diagnostic mockup. Conservative veneer preparations were performed on the diagnostic mockup with horizontal depth grooves using a diamond bur (801 FG, Jota, Rüthi, Switzerland) followed by tooth preparation that was constantly checked with reduction guides (Figure 5). 

**Figure 6:**
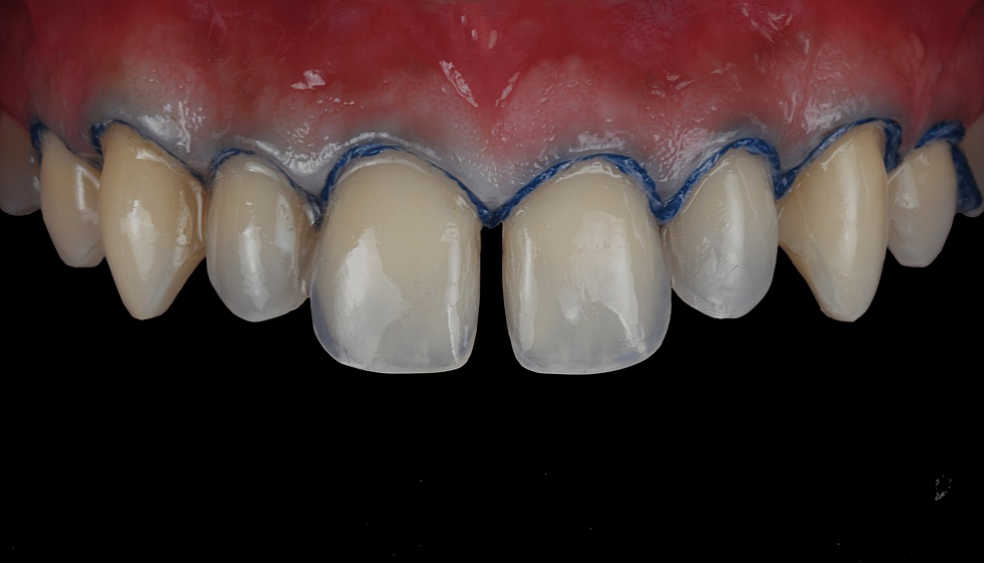
Final preparations and cord packing before final impression

Final tooth preparations were polished with polishing discs (Sof-Lex XT Discs, 3M, Saint Paul, MN, USA) and polishing wheels (Polishing Kit 1921, Jota). A single retracting cord (#00 retraction cord plain knitted, Ultrapak, Victoria, Australia) was placed and a final impression was made with polyvinyl siloxane material (Virtual 380, Ivoclar Vivadent) in heavy- and light-body consistency (Figure 6). 

**Figure 7:**
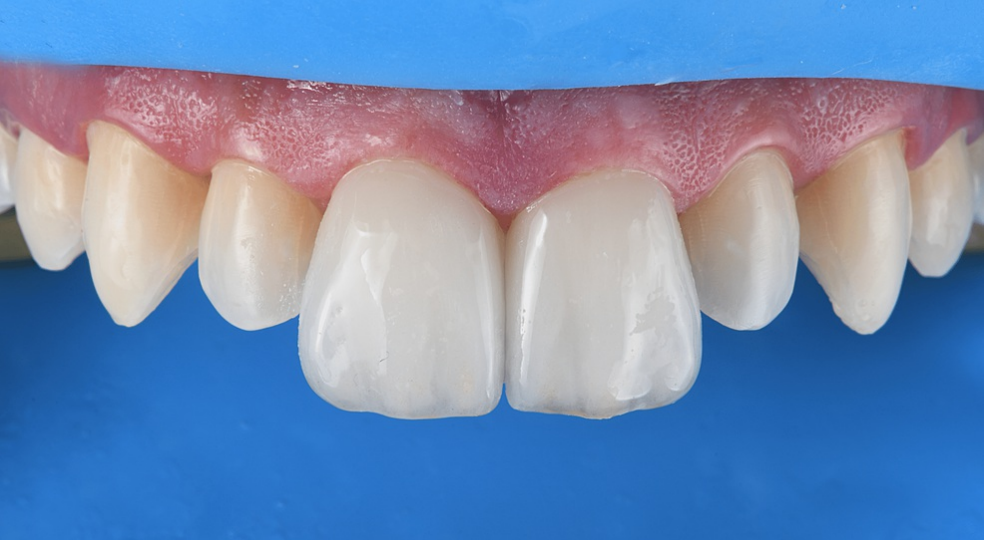
Bonding procedure under rubber dam

The final impression was poured out with type IV stone (Fujirock, GC America, IL, USA) and cross-mounted in the articulator with the mandibular diagnostic cast. Feldspathic porcelain veneers were hand-crafted following the diagnostic wax-up contours. A dry try-in of the restorations was performed in the patient’s mouth in order to evaluate margins, contours, and shade. The patient approved the try-in and requested to cement the final prosthesis. Tooth isolation prior to bonding procedures was provided with a rubber dam (dental dam, Nic Tone, Bucharest, Romania) and holding clamps on molars #3 and #14. Teeth were sandblasted with 29-micron aluminum oxide (AquaCare, Velopex, London, UK) followed by tooth treatment with 37% phosphoric acid (Total Etch, Ivoclar Vivadent) for 15 seconds and rinsed and air dried, followed by primer and adhesive (Variolink Esthetic LC, Ivoclar Vivadent) application, post which the ceramic was placed and excess was removed. Light curing was done for 20 seconds on facial, incisal, palatal, mesial, and distal (Figure 7). 

**Figure 8:**
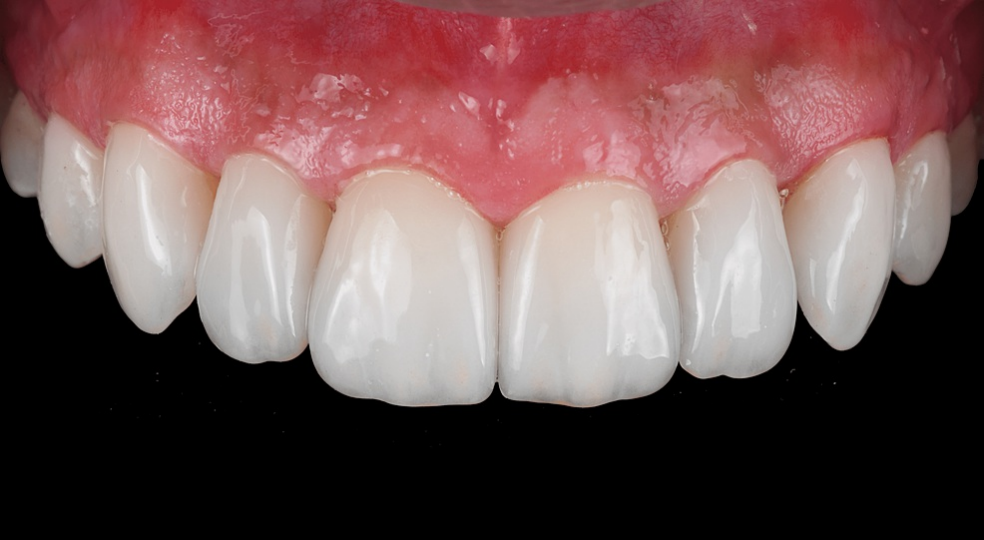
Final restorations

**Figure 9:**
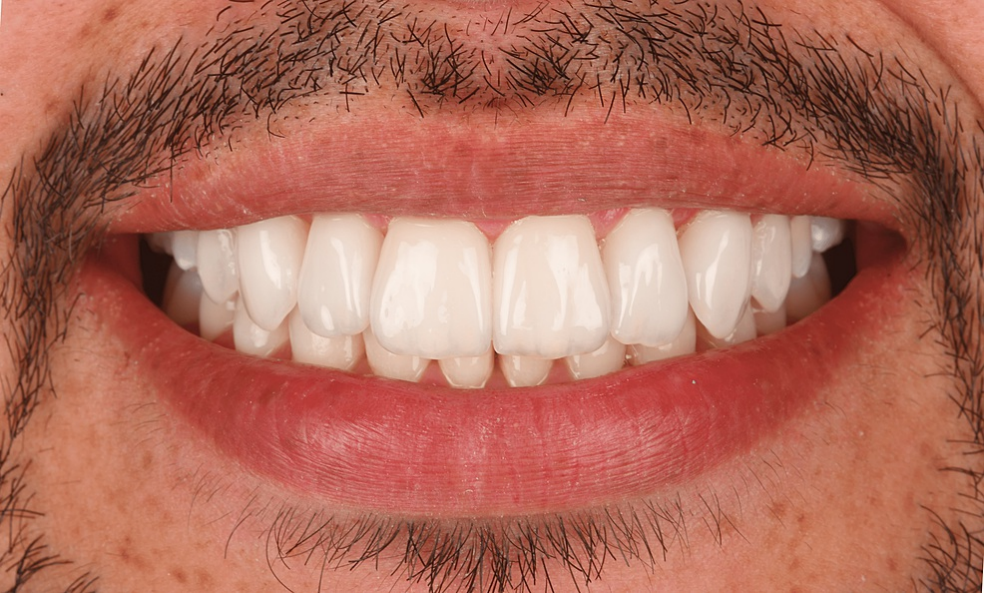
The patient's smile after ceramic restoration

Then, the rubber dam was removed, the occlusion was checked, adjusted and restorations were polished (Dialite Feather Lite, Brasseler USA, GA, USA) with a polishing paste (Dialite Intraoral Polishing Paste, Brasseler USA). The patient was pleased with the overall appearance of the ceramic restorations (Figure 8 and Figure 9). 

**Figure 10:**
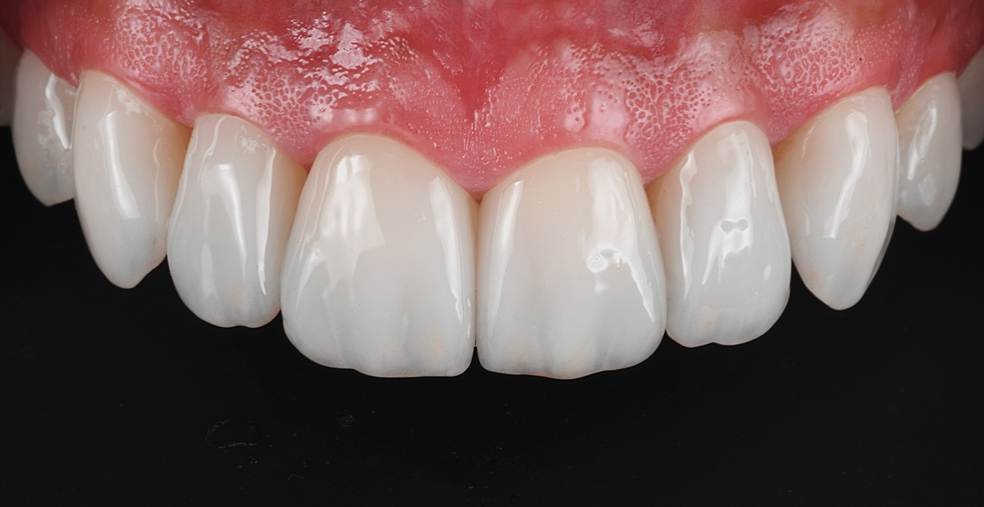
Intraoral picture of the patient's restorations and periodontal tissues at the five-year follow-up

The patient displayed a good condition of the restorations and the periodontal tissues at the five-year follow-up (Figure 10). 

**Figure 11:**
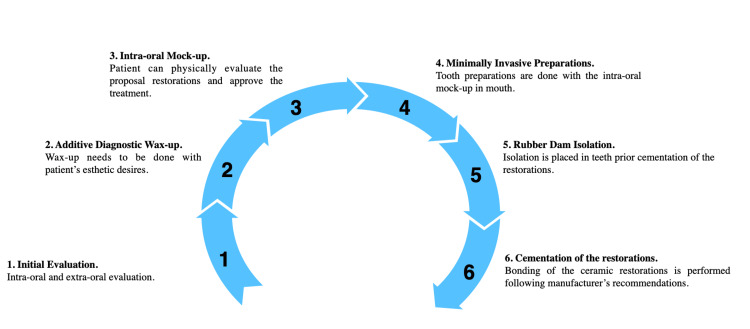
Clinical workflow for minimally invasive veneer restorations

Overall, the described initial diagnostic additive wax-up and intraoral mockup provided a predictable end result (Figure 11). 

## Discussion

Dental restorations in the esthetic zone are driven by factors related to teeth and soft tissues but also the patient’s expectations. A proper initial examination is required for the desired outcome with minimally reduced preparations. This examination should start with an extraoral assessment including the facial skeletal, smile line, the number of teeth and amount of periodontal tissues on display, lips, phonetics, and esthetic requests from the patient. These exams can detect other patient desires such as shifting of the midline. The smile evaluation should include the entire lower third of the face that extends from the base of the nose to the menton because disproportional tooth or veneer shapes related to the size of lips, commissures, and chin may result in a negative perception of the smile by the patient [14]. An extraoral evaluation was provided to this patient and acceptable parameters were found, however, the smile assessment revealed spaces between teeth and uneven incisal edges for both central and both lateral incisors. This patient presented a stable occlusion with no missing teeth and without interferences during protrusion and lateral movements. As a result, the patient was an ideal candidate for anterior restorations. The clinical features needed to achieve minimally invasive veneer preparations are summarized in Table 1. 

**Table 1:** Clinical features needed to achieve minimally invasive veneer preparations

Achievable Factors	Unachievable Factors
Additive diagnostic wax-up is possible without modifying the cast	Additive diagnostic wax-up is not possible and the cast needs to be modified
Teeth in ideal position following the arch form	Teeth in malposition (crowded/rotated/tilted/flared out).
Competent lip	Potentially competent and/or incompetent lip
No caries present	Caries
No changes or only slight changes in shade are required (one shade)	Major changes in shade are needed with the restoration (two or more tooth shades)
Light color dentin	Dark dentin
No developmental disorders	Developmental disorders that cause stained teeth (e.g.: amelogenesis imperfecta)
Stable occlusion	Edge-to-edge occlusion in anterior teeth
*If a patient presents one unachievable factor, the minimally invasive veneer preparations will be compromised but traditional veneer preparation may be a potential treatment.	

Tooth proportions are key for a natural-looking smile, especially the size and shape of front teeth that contribute to a harmonious dentition [15]. Some authors have reported that specific width and length ratios from central incisors to first premolars are needed for a beautiful smile [16]. In the present report, the patient presented with non-ideal incisal edges, non-ideal width and length ratios, and spaces between teeth. Therefore, clinicians needed to perform diagnostic wax-up with modified ratios to close spaces and obtain close to ideal ratios. Diagnostic wax-ups can be traditional or additive. The traditional wax-up requires cast modifications and typically is needed for patients with tilted, rotated, extruded, protruded, or present restorations that need to be changed. Traditional wax-up with cast modification limits the clinician and patient because diagnostic mockup is not possible to preview the proposed restorations. Additive diagnostic wax-up is possible whenever the lips are competent, occlusion is stable, and teeth are correctly aligned in the arch [17]. It is important to perform the diagnostic wax-up following the patient’s desires and present the desired result for patient satisfaction. The patient herein had all teeth aligned in the arch shape, lips were competent and the spaces between the teeth did not require cast modifications to place wax. Therefore, additive wax-up was possible and it closed the space between the teeth and increased the length of teeth to have a more ideal and natural-looking shape. 

The diagnostic mockup is provided to the patient with a silicone matrix that is fabricated from the diagnostic wax-up. The silicone index is filled up with temporary resin or bis-acryl material to transfer to the patient’s mouth [18]. A diagnostic mockup is an important tool as it provides a physical evaluation of the proposed restorations to both the patient and clinician. Since the diagnostic wax-up is made following the patient’s esthetic concerns, the mockup will fulfill the patient’s satisfaction, however, adjustments can also be done in the diagnostic mockup to fulfill any last-minute esthetic desires. Diagnostic mockup is a reversible procedure, therefore the patient and clinician can cancel or postpone the irreversible restorative procedure with tooth preparations [19]. Once the diagnostic mockup fulfills a patient’s esthetic demands, photographs of it are taken by the dental technician to help fabricate the final prosthesis by copying the diagnostic wax-up and the photos. Our patient was fully satisfied with the diagnostic mock-up and approved the proposed restorations. Photographs were taken to keep a record along with the diagnostic wax-up for the dental technician to hand-craft the veneer restorations. 

Some drawbacks of the conventional wax-up and diagnostic mockup used here include a significant investment of time, difficulties in making small changes for indecisive patients, and inaccuracies at the incisal edge and cervical margin. Digital techniques like digital scanners and 3D-printed mockups open the door for more time efficiency. A fully digital workflow may be more reliable than conventional techniques and makes use of planning software such as digital smile design. However, significant monetary and time investments are needed to use these techniques, which may not be feasible for a general dentist. 

The present report described how an additive diagnostic wax-up and intraoral mockup provide relevant information regarding the possibility of minimally invasive veneer restorations for patients. If teeth are properly aligned in the arch form, and without overlap, then the additive diagnostic wax-up can be performed. The intraoral mockup is a reversible procedure that gives an intraoral evaluation of the proposed restorations before any irreversible procedure. Clinicians following the described procedures can successfully diagnose and provide minimally invasive veneer restorations. 

## Conclusions

Performing additive wax-ups can provide information on the amount of tooth reduction needed prior to tooth preparations. In tandem, diagnostic mockups provide the patient with a physical evaluation of the proposed restorations before proceeding with irreversible procedures. Diagnostic wax-up and mockup should be considered the driving tools for minimally invasive restorations in the esthetic zone.
